# Characterisation of niosome nanoparticles prepared by microfluidic mixing for drug delivery

**DOI:** 10.1016/j.ijpx.2022.100137

**Published:** 2022-11-01

**Authors:** Mohammad A. Obeid, Ibrahim Khadra, Alaa A.A. Aljabali, Haneen Amawi, Valerie A. Ferro

**Affiliations:** a Department of Pharmaceutics and Pharmaceutical Technology, Faculty of Pharmacy, Yarmouk University, P.O.BOX 566, Irbid 21163, Jordan; b Strathclyde Institute of Pharmacy and Biomedical Sciences, University of Strathclyde, 161 Cathedral Street, G4 0RE Glasgow, United Kingdom; c Department of Pharmacy Practice, Faculty of Pharmacy, Yarmouk University, P.O.BOX 566, Irbid 21163, Jordan

**Keywords:** Niosomes, Drug delivery, Microfluidic mixing, Atenolol, Drug release

## Abstract

Lipid nanoparticles have gained much attention due to their potential as drug delivery systems. They are safe, effective, and be targeted to particular tissues to deliver their payload. Niosomes are one type of lipid nanoparticles that comprise non-ionic surfactants which have proven to be effective due to their stability and biocompatibility. Different manufacturing processes have been reported for niosome preparation, but many of them are not scalable or reproducible for pharmaceutical use. In this study, microfluidic mixing was used to prepare niosomes with different lipid compositions by changing the type of non-ionic surfactant. Niosomes were evaluated for their physicochemical characteristics, morphology, encapsulation efficacy, release profiles of atenolol as a model hydrophilic compound, and cytotoxic activities. Microfluidic mixing allows for particle self-assembly and drug loading in a single step, without the need for post-preparation size reduction. Depending on the lipid composition, the empty particles were <90 nm in size with a uniform distribution. A slight but not significant increase in these values was observed when loading atenolol in most of the prepared formulations. All formulations were spherical and achieved variable levels of atenolol encapsulation. Atenolol release was slow and followed the Korsmeyer-Peppas model regardless of the surfactant type or the percentage of cholesterol used.

## Graphical abstract

**Figure:**
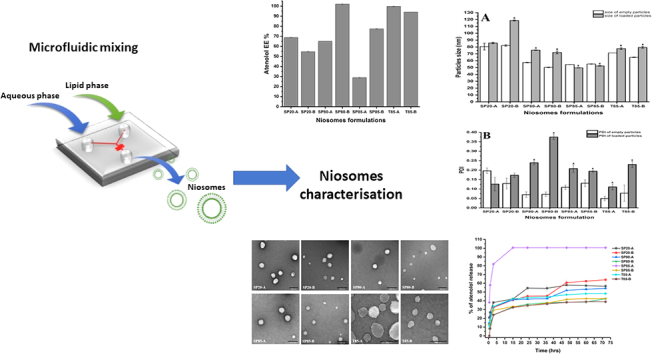

## 1 Introduction

Niosomes are lipid-based nanoparticles that are generated by the self-assembly of non-ionic surfactants and other lipid materials into a spherical bilayer structure. The bilayer makes these particles versatile, where hydrophilic drugs can be encapsulated into the core aqueous compartment, while hydrophobic drugs can be embedded into the lipid membrane (Muzzalupo and Mazzotta, 2019; Obeid et al., 2021a). This structural confirmation makes niosomes an excellent drug delivery system. Different types of non-ionic surfactants can be used to prepare niosomes, such as Span, Tween, and Brij surfactants. Cholesterol may also be included to increase the rigidity and reduce the permeability of the membrane bilayer of the generated niosomes (Taymouri and Varshosaz, 2016; Yasamineh et al., 2022).

Niosome production for therapeutic drug delivery aims to achieve predictable, controllable particle size distributions, and controlled release of the loaded drug over prolonged periods. Different methods have been reported for the production of niosomes, including thin-film hydration, heating, freeze-thaw cycling, reverse phase evaporation, detergent depletion, and injection methods (Obeid et al., 2017a). However, these traditional bulk mixing methods end up producing heterogeneous niosomes, which are poly-disperse in terms of particle size and lamellarity (Marianecci et al., 2014). These limitations can be overcome using microfluidic mixing techniques that overcome the traditional preparation methods' heterogeneity and reproducibility problems (Obeid et al., 2019). Microfluidic mixing is based on mixing fluids within small channels of 10–100 mm in diameter (Whitesides, 2006). The application of microfluidic mixing in the preparation of niosomes for drug delivery purposes results in controllable small and monodisperse vesicles with high stability profiles (Obeid et al., 2020a).

Niosome preparation with microfluidic mixing starts with dissolving the non-ionic surfactant, cholesterol, and other components into an organic solvent such as ethanol and then mixing this lipid phase with an aqueous phase such as phosphate buffer. During the mixing process, several factors should be controlled, such as flow rate ratios (FRR) between the aqueous and the lipid phases, the total flow rates (TFR) of both phases, and the mixing temperature (Carugo et al., 2016).

The ability to control the characteristics of the generated particles such as the particles size, the size distribution, and encapsulation efficiency is crucial factor in the success of nanoparticles preparation with microfluidic mixing as these characteristics will influences the *in vivo* performance (Lo et al., 2010). Several factors should be controlled during the preparation of lipid nanoparticles with microfluidic mixing in order to achieve the required *in vivo* effects. In our previous work, we have investigated the effects of the TFF, FRR, the type of aqueous media and the lipids concentration on the characteristics of the generated particles (Obeid et al., 2017b). In the present work, we aim to examine the effects of using different types of non-ionic surfactants for the production of niosomes by microfluidic mixing on the characteristics of the generated particles such as the particles size and morphology, encapsulation efficiency and release profile of atenolol as a hydrophilic model drug along with the formulations toxicities. Different types of non-ionic surfactants were examined, such as Sorbitan monolaurate (Span 20), Sorbitan monooleate (Span 80), Span 85 (SP85), and Tween 85 (T85) at different ratios with cholesterol and the niosomes prepared by mixing the lipid phase with the aqueous phase through a microchannel. The prepared niosomes were characterised in terms of their particle size and polydispersity index (PDI). Transmission electron microscope (TEM) studies were performed in order to assess the morphology of the prepared niosomes. The ability of the niosomes to act as a drug delivery system and encapsulate the model hydrophilic drug atenolol was evaluated. This was carried out by measuring the atenolol encapsulation efficiencies and the release profile of atenolol from the different niosome formulations when stored at 37 °C for 72 h to assess if it was possible to achieve a desired controlled release profile. Finally, the cytotoxicity of the prepared niosome formulations was evaluated on a murine macrophages (RAW 264.7) and human breast cancer (T47D) cell line. Here, we demonstrated the novelty and the feasibility of niosome preparation by microfluidic mixing in seconds, through rapid and controlled mixing of two miscible phases (lipids dissolved in alcohol and an aqueous medium) in a microchannel, without the need of a size reduction step, as required for the conventional methods. Moreover, hydrophilic drugs can be encapsulated in the generated niosomes in an attempt to generate slow release formulations for more efficient therapeutics with less frequency of administration. This can help the pharmaceutical industries in the large-scale production of these nanoparticles-based formulations by optimising all the preparation parameters.

## 2 Materials and methods

### 2.1 Materials

Span 20 (SP20), Span 80 (SP80), SP8, T85, Cholesterol (CH), phosphate-buffered saline tablets (PBS, pH 7.4), cellulose membrane dialysis tubing (molecular weight cut-off 14,000 kDa), atenolol, and ethanol were all obtained from Sigma-Aldrich, UK.

### 2.2 Preparation of niosomes

Niosomes were prepared with microfluidic mixing using a NanoAssemblr™ (Benchtop, Precision NanoSystems Inc., Vancouver, Canada) as reported previously by Obeid et al. (Obeid et al., 2017a).

The NanoAssemblr™ adopted a mixing channel with staggered herringbone structures with two inlet streams for the lipid and aqueous phases and one outlet for the prepared niosome formulations. The staggered herringbone structure used in this microchannel produced a rotational flow between the two phases, resulting in wrapping the two phases around each other with a chaotic flow profile for efficient and faster mixing (Belliveau et al., 2012). The purpose was to enhance the self-assembly of the lipid components into a bilayer structured vesicle.

To prepare the niosomes, ethanolic solutions of the chosen non-ionic surfactants were mixed with CH at different molar ratios (lipid phases), as detailed in Table 1. Each lipid phase was then injected into the microchannel apparatus to be mixed with PBS, with and without atenolol (aqueous phase). The mixing process was achieved through computerised syringe pumps. The FRR and the TFR between the two phases were controlled to change the characteristics and the size of the prepared nanoparticles. Here, the two phases were mixed at an FRR of 3:1 between the aqueous and lipid phases, and both phases were injected at a TFR of 12 ml/min rate. The system was continuously checked for any leakage during the particles preparation.

**Table 1:** Composition of the different niosome formulations.

Sample ID	Composition	Molar ratio
SP20-A	SP20:CH	50:50
SP20-B	SP20:CH	70:30
SP80-A	SP80:CH	50:50
SP80-B	SP80:CH	70:30
SP85-A	SP85:CH	50:50
SP85-B	SP85:CH	70:30
T85-A	T85:CH	50:50
T85-B	T85:CH	70:30

### 2.3 Niosome physicochemical characterisation by dynamic light scattering (DLS)

The generated particle size and PDI were measured by DLS using a Zetasizer Nano-ZS (Malvern Instruments Ltd., UK). The measurements were carried out in triplicate under the conditions of 25 °C, electrical field 13.89 V cm, refractive index 1.330, and voltage 5 V. The different niosome samples, with and without atenolol were diluted 1/10 using PBS at pH 7.4, and the z-average particle size (*Z*-average) and PDI were recorded.

### 2.4 Morphological analysis using a transmission electron microscope (TEM)

Carbon-coated copper grids (400 mesh, agar scientific) were glow discharged in the air for 30 s. A volume of 5 μl from each formulation was drop-cast on the grids and was then negatively stained using a 1% (*w*/*v*) aqueous solution of uranyl acetate. Each sample was allowed to air dry in a dust-free environment before imaging using a JEOL JEM-1200 EX TEM, operating at 80 kV (JEOL, Tokyo, Japan). TEM images for each formulation were collected from random regions of the grid.

### 2.5 Determination of niosome encapsulation efficiency (EE)

The dialysis method was used to remove free, unencapsulated atenolol before determination of its EE in the niosomes. After preparing atenolol-loaded niosomes, each formulation was transferred to dialysis tubing (14,000 kDa cut-off), and both ends were sealed. Each tube was dialyzed against 10× PBS to maintain sink conditions at ambient temperature with continuous stirring using a magnetic stirrer (500–1000 rpm). At different time intervals, 1 ml was sampled from the PBS dialysis media, and the atenolol concentration was determined by measuring the UV absorbance at λ = 276 using a ThermoSpectronic spectrophotometer (Thermo Fisher Scientific, UK). The dialysis was carried out until no more atenolol was detected in the dialysis media, and a constant atenolol concentration was achieved. After removing free atenolol, encapsulated atenolol was released from 100 μl of each niosome formulation using 100% methanol. The released atenolol concentration was then determined spectrophotometrically at λ = 276 nm.

The EE of atenolol was determined according to the following equation:

(1) $$\mathrm{EE}\%=\left(\text{Amount of drug encapsulated}/\text{initial drug added}\right)*100\%$$

The experiments were performed in triplicate, and the average EE% ± SD was reported.

### 2.6 Atenolol release profiles

Following the removal of free atenolol, 2 ml of each niosome formulation was dialyzed against 10× PBS with continuous stirring for 72 h and stored at 37 °C in a controlled temperature room. At specific time intervals (0.5, 1, 3, 12, 24, 36, 48, 64, and 72 h), 1 ml from the dialysis media was taken, and the amount of released atenolol was determined by measuring the absorbance at λ = 276 nm. The released atenolol concentration was determined using an atenolol standard curve prepared at concentrations from 3.25 to 835 μg/ml. At each time point, each sampling was replaced with fresh PBS preheated at 37 °C in order to maintain the sink conditions. To evaluate the impact of the dialysis membrane on the observed drug release kinetics, the release profile of free atenolol was evaluated by adding atenolol solution at concentration of 1 mg/ml in the dialysis bag and dialyzed against 10× volume of PBS with continuous stirring for 72 h. At each time point, samples from the dialysis media were taken and the amount of released atenolol was determined by measuring the absorbance at λ = 276 nm.

### 2.7 The release kinetics of atenolol

To determine the release kinetics of atenolol for each niosome formulation, the *in vitro* drug release data were fitted with different release kinetic models and evaluated to understand the kinetics of drug release. These included a zero order model, a first order model, Higuchi's model, Korsmeyer-Peppas model, and Hixon-Crowell's model (Gouda et al., 2017) as follows:

1. The zero-order rate equation is defined as:

(2) $$C_{0}-C_{t}=K_{0}t$$

Where C_t_ is the amount of drug released at time t, C_0_ is the initial concentration of drug at time *t* = 0, K_0_ is the zero-order rate constant.

2. First order model is defined as:

(3) $$\log\;C=\log\;C_{0}-K_{1}\;t/2.303$$

where C_0_ is the initial concentration of the drug, C is the percent of drug remaining at time t, and K_1_ is the first order rate equation expressed in time^−1^ or per hour.

3. The Korsmeyer and Peppas model is defined as:

(4) $$F=M_{t}/M=K_{m}t^{n}$$

where F is a fraction of drug released at time t, M_t_ is the amount of drug released at time t, M is the total amount of drug in dosage form, K_m_ is the release rate constant, and n is the release exponent.

4. Hixon-Crowell's model is defined as:

(5) $${Q_{0}}^{1/3}–{Q_{t}}^{1/3}=\mathrm{KHCt}$$

where Q_0_ is the initial amount of drug in the niosomes, Q_t_ is the remaining amount of drug in the niosomes at time t, and KHC is the Hixson-Crowell release constant.

From the above-mentioned models, the most suitable kinetics model for the atenolol release profile for each niosome formulation was selected on the basis of the best fit, *i.e.*, the model with the highest regression coefficient of correlation (r^2^) (closer to 1) indicated the release model of atenolol from each formulation.

### 2.8 Formulations cytotoxicity

The *in vitro* cytotoxicity of various niosome formulations encapsulated atenolol, empty niosomes and free atenolol on a macrophages (RAW 264.7) and breast cancer (T47D) cell line was investigated using a [(3- (Method 4, 5-dimethylthiazole-2-yl) -2) 5- Diphenyl-tetrazolium bromide (MTT) assay (Meerloo et al., 2011). For this purpose, both RAW 264.7 and T47D cell lines were cultured as an adherent monolayer in culture flasks in Dulbecco's modified Eagle's medium (DMEM) supplemented with 4.5 g of glucose, 10% (*v*/v) foetal bovine serum (FBS), and 1% (*w*/*v*) penicillin/streptomycin. The cells were then seeded in 96-well plates and incubated in a humidified incubator containing 5% CO_2_ at 37 °C for 24 h. The cells were treated with different concentrations of each niosome formulations, with and without atenolol in order to evaluate any toxic effects of the prepared formulations. Briefly, cells were treated with MTT and re-incubated for 4 h and then the media were carefully withdrawn and formed formazine crystals were dissolved with DMSO and the absorbance was measured at 570 nm using a microplate reader (synergyMx BioTek, USA). Cell viability was expressed as a percentage of the absorbance obtained for untreated cells.

### 2.9 Statistical analysis

One-way analysis of variance (ANOVA) was used to assess statistical significance. A Tukey's multiple comparison test and *t*-test were performed for paired comparisons. The statistical analysis was performed using Minitab software version 17. A *p* value <0.05 was considered statistically significant.

## 3 Results and discussion

### 3.1 Niosome physicochemical characterisation

In this research, the effect of changing the type and percentage of the non-ionic surfactant on the physicochemical characteristics of niosomes prepared by microfluidic mixing was investigated. The evaluation of the prepared niosomes was performed by considering the size, size distribution, and EE of the antihypertensive drug atenolol as a model hydrophilic drug. The goal of designing and developing an effective drug delivery system is to achieve a proper drug loading and desired release properties with a high half-life and low toxicity (Shah et al., 2020; Obeid et al., 2021b).

**Fig. 1:**
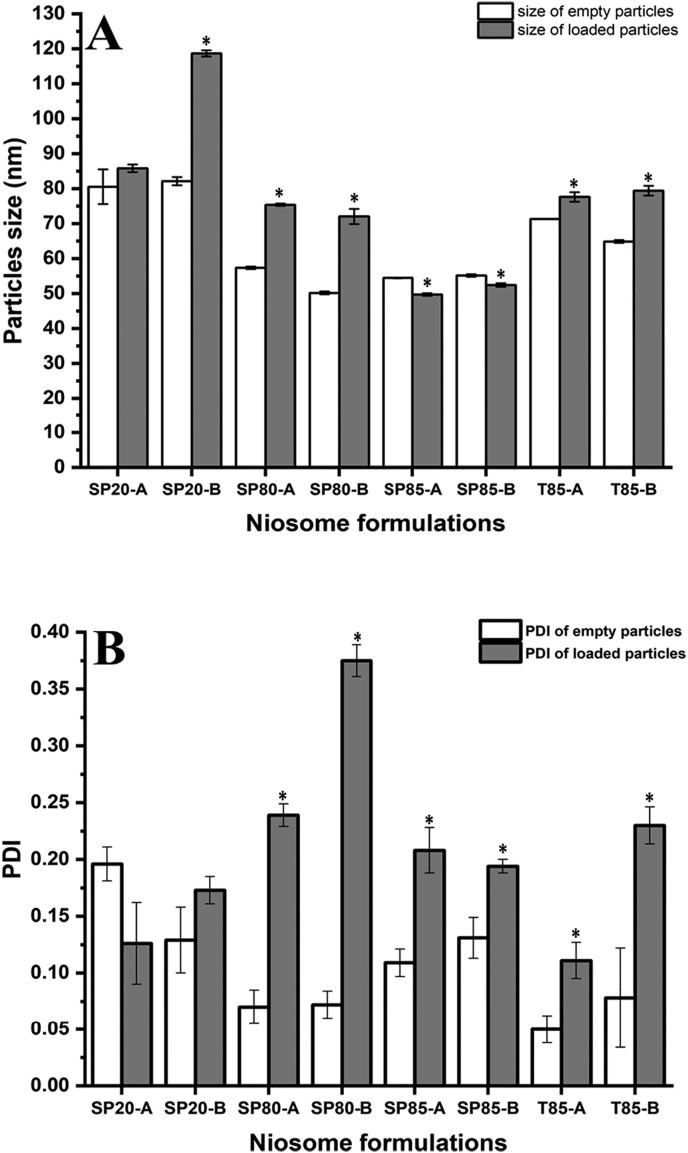
Size and PDI values for different niosome formulations before and after loading with atenolol. *Represents a significant difference before and after atenolol encapsulation. Results represent mean ± SD of triplicate readings.

Fig. 1 shows the particle size and PDI values of the prepared niosome formulations before and after atenolol encapsulation.

Empty niosomes had different particle sizes depending on the type of non-ionic surfactant used and the CH percentage in each formulation. This can be attributed to the differences in the hydrophilic-lipophilic balance (HLB) value of each surfactant. Moreover, the size of most of the formulations increased slightly with atenolol encapsulation. This was expected as atenolol is a hydrophilic molecule and is likely to be encapsulated into the hydrophilic compartment of the niosome nanoparticles, which might result in a slight increase of the particle size. However, this was not the case in niosomes prepared using SP85, where the encapsulation of atenolol resulted in a slight decrease in the particle size compared to the empty particles. This can be attributed to differences in each surfactant's critical packing parameter, including SP85, which might result in higher atenolol packing than the empty particles when using SP85 as the surfactant (Bhardwaj et al., 2020).

In terms of particle distribution, atenolol encapsulation resulted in a significant (*p* < 0.05) increase in the PDI values of all niosome formulations except in formulation SP20-A, where the encapsulation of atenolol resulted in a slight and non-significant (*p* > 0.05) decrease in the PDI value (Fig. 1B). However, regardless of this increase in the PDI values, all the loaded niosome formulations were monodisperse in distribution as the maximum PDI value was for SP80-B formulation and was <0.40. However, in all cases, these results confirms what has been previously reported in the literature about the microfluidic mixing production of lipid nanoparticles where this method was shown to produce small sized nanoparticles with low distribution in a single production step (Belliveau et al., 2012). Although the niosomes composition affects the generated particles size and size distribution, still all the generated formulations were small in size (<200 nm) with monodisperse characteristics. This confirms the suitability of microfluidic mixing for the industrial scale production of such nanoparticles with high reproducibility (Tomeh et al., 2022). Similar results about the effects of changing the niosome compositions on the generated particles size were reported by Nowroozi *et al*, where they prepared different niosome formulations using the thin film hydration method followed by the use of various size reduction techniques such as extrusion and probe sonication (Nowroozi et al., 2018).

### 3.2 Niosome morphology

**Fig. 2:**
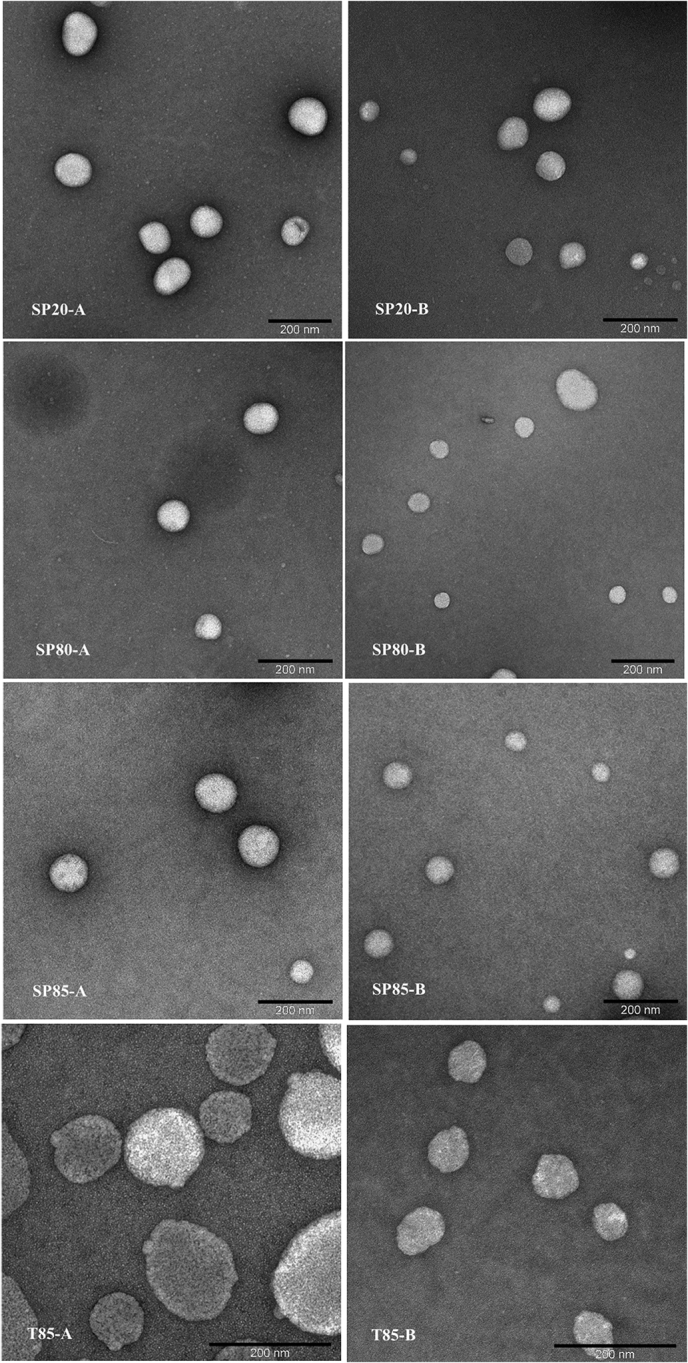
TEM images for the different empty niosome formulations.

The morphology of the different empty niosome formulations can be seen in the TEM images (Fig. 2). The niosomes were spherical, with particle size distribution comparable to the DLS results. The size of the particles with the TEM images appears different to the results obtained from the DLS, this is because the samples were dried before TEM analysis. This phenomena has been noticed in our previous work when the niosomes samples dried and examined under the transmission electron microscopy (Obeid et al., 2020b) and the atomic force microscopy (Obeid et al., 2020c). In this work, the morphology of the loaded niosomes was not investigated since atenolol is a hydrophilic molecule and will be loaded by encapsulation into the aqueous core of the niosomes. Therefore, the surface of the loaded niosomes is thought to be the same as the empty niosomes.

### 3.3 Encapsulation of atenolol

**Fig. 3:**
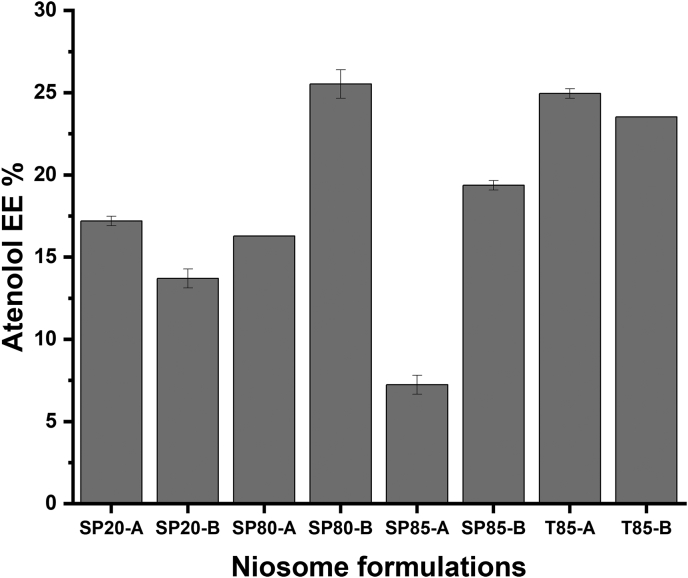
The percentage of atenolol EE in different niosome formulations. Results represent mean ± SD of triplicate readings.

Atenolol was used as a model hydrophilic drug to be encapsulated at a starting concentration of 10% (*w*/w) compared to the total lipid concentration used in the formulation. Atenolol was dissolved in the aqueous phase of the microfluidic mixing while the lipids were dissolved in ethanol. By the time the lipids self-assemble into a bilayer structure, some percentage of atenolol will be encapsulated into the aqueous compartment of the niosomes (Correia et al., 2017; Aljabali and Obeid, 2020). The percentage atenolol encapsulation (Fig. 3) indicates clearly that the type of surfactants used will significantly affect the percentage of atenolol encapsulation. This variation in drug loading is related to the differences in the HLB value of each surfactant that influences the drug EE of the particular niosomes (Bhardwaj et al., 2020; Abdelkader et al., 2014; Alyamani et al., 2019; Aljabali et al., 2019). This has been reported extensively in the literature where changing the type of surfactant or phospholipid in liposome preparations will significantly affect the percentages of drug loading (Obeid et al., 2020b; Gregoriadis, 1973; Jain and Jain, 2016; Gonzalez Gomez and Hosseinidoust, 2020; Maritim et al., 2021; Aljabali et al., 2020). The highest atenolol EE was observed when using SP80 and T85 as the non-ionic surfactants for the niosome preparations (∼25%) and the lowest atenolol EE was observed when SP85 was used (∼7%). El-assal et al. (2017) reported preparing different pro-niosome formulations for atenolol encapsulation using span 20, 40, 60, and 80. They reported that the type of surfactant affected both the physicochemical characteristics of the prepared particles and the percentage of atenolol entrapment, where the latter was highly dependent on the surfactant type and the aqueous media used in the particle preparation (El-Assal, 2017).

### 3.4 Atenolol release profiles

**Fig. 4:**
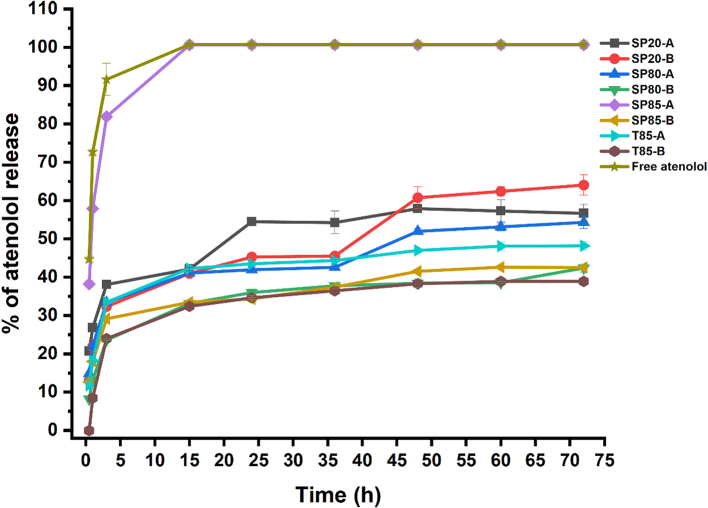
The rates of atenolol release from different niosome formulations over 72 h at 37 °C. Results represent mean ± SD of triplicate readings.

Following the preparation and encapsulation of different atenolol-loaded niosomes, the release profile of atenolol was examined to determine whether controlled release profiles of the drug could be achieved. The rates of atenolol release from the different niosome formulations over 72 h were measured (Fig. 4). Formulation SP85-A resulted in the fastest rate of atenolol release, where almost all the encapsulated atenolol was released in the first 15 h. Niosome formulations T85-B and SP80-B resulted in the slowest rates of atenolol release throughout the study. The other formulations showed various rates of atenolol release, which indicates that the type of non-ionic surfactant used in the microfluidic mixing preparation will significantly affect the rate of atenolol release. The release of free unloaded atenolol from the dialysis membrane was faster than the atenolol release from any of the niosome formulations. This indicates that the observed atenolol release profiles from the different niosome formulations was due to the niosome encapsulation and not an impact of the dialysis membrane. In all the niosome formulations, the atenolol release showed a two-step process, a relatively rapid burst drug release phase, which might be due to the release of the drug from the outer surface of the niosomes followed by a slower drug release as a result of the penetration of the drug through the niosome bilayer membrane. This can be useful in reducing the frequency of atenolol administration by administering niosmes loaded with atenolol with slow-release profiles.

### 3.5 Atenolol release kinetic model

To determine the release kinetics of atenolol based on the release data, the *in vitro* atenolol release data from each niosome formulation were fitted to different kinetic models and linear forms of each kinetic model were drawn for the niosome formulations. The release model with a linear regression coefficient (closest to 1) represents the kinetic model of atenolol release from that formulation (Temprom et al., 2022). The regression coefficient (R^2^) values for each kinetic model are presented in Table 2. The kinetics release data results revealed that the release of atenolol from all the prepared niosome formulations was best explained by the Korsmeyer-Peppas model regardless of the non-ionic surfactant type or the percentage of CH used. In the Korsmeyer-Peppas model, the n value is used to characterize different diffusion release mechanisms as given in tabular form for cylindrical shaped matrices. In these results, the n values were <0.5 for all the niosome formulations.

**Table 2:** The linear regression coefficient (R^2^) values for different kinetic models of atenolol release from various niosome formulations.

Release model	Zero order	First order	Korsmeyer-Peppas		Hixson-Crowell
	R^2^	R^2^	R^2^	n	R^2^
SP20-A	0.6439	0.1385	0.9763	0.1852	0.3382
SP20-B	0.8063	0.1496	0.9768	0.2675	0.4467
SP80-A	0.7064	0.1424	0.9740	0.2091	0.3742
SP80-B	0.6803	0.1487	0.9706	0.2411	0.4073
SP85-A	0.7060	0.1316	0.9676	0.1389	0.2614
SP85-B	0.6614	0.1407	0.9709	0.1945	0.3528
T85-A	0.6052	0.1429	0.9506	0.2042	0.3572
T85-B	0.6800	0.1598	0.9528	0.2773	0.4538

### 3.6 Niosomes cytotoxicity

**Fig. 5:**
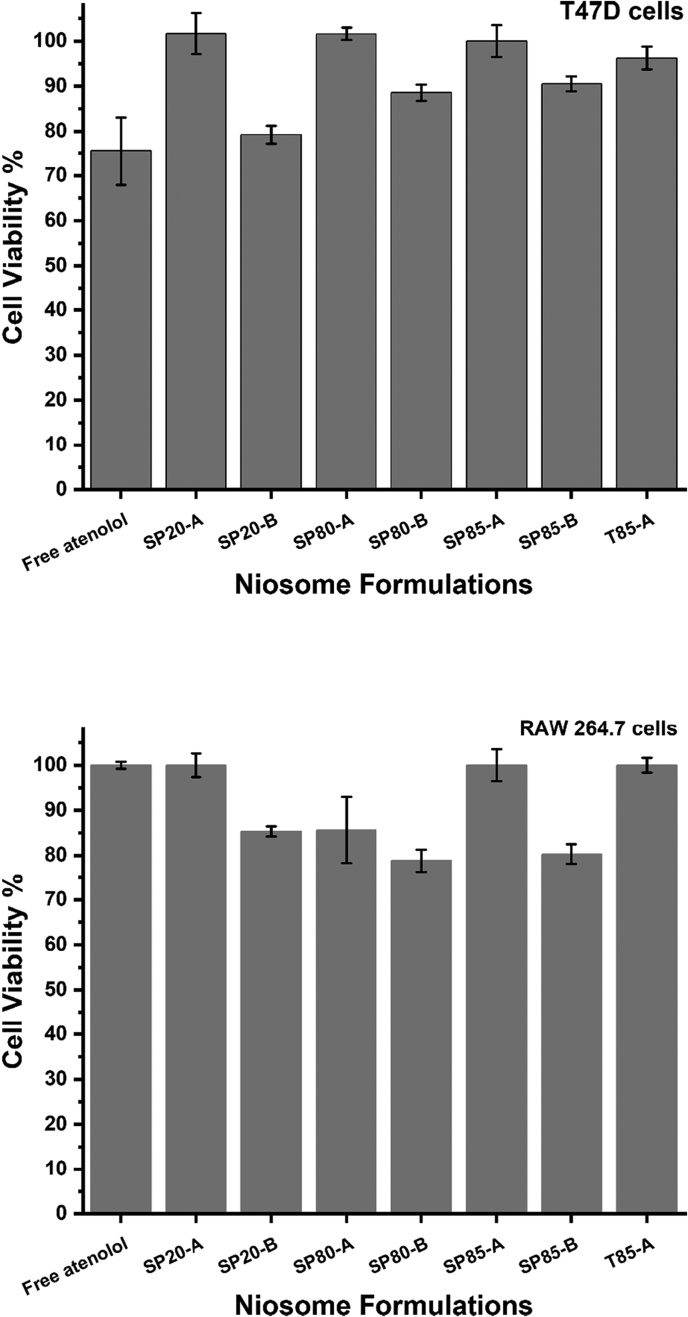
The percentages of T47D and raw macrophages viabilities when treated with 250 μg/ml niosomes loaded with atenolol. Results represent mean ± SD of triplicate readings.

In order to evaluate the cytotoxic effects of the prepared niosomes formulations, both a macrophages and cancer cell line were treated with all the formulations, with and without atenolol, starting from a niosome concentration of 250 μg/ml and then serially diluted. Results showed that the T47D cell viabilities were >80% for all formulations (Fig. 5). Moreover, T47D cells treated with empty niosomes also showed >80% viability for all formulations (data not shown). Similarly, the RAW 264.7 macrophages also showed >80% viability for all formulations (Fig. 5). It was noted that the cell viabilities for formulations A that had equal molar ratios between the surfactants and CH were higher than the viabilities of formulations B that had a higher surfactant percentage and lower CH (Fig. 5). These results show that the surfactants used here are relatively not toxic and these nanoparticles can be used safely to encapsulate and improve the kinetic profile of atenolol. This was comparable with the cytotoxicity results reported in the literature for other niosomes (Obeid et al., 2021b; Gebril et al., 2022; Sangboonruang et al., 2021). For example, in the work of Hajizadeh *et al*, niosome prepared by film hydration technique with Span 40: Tween 40: cholesterol at molar ratio of 35:35:30 was shown to have low toxicity and good safety for healthy cells (Hajizadeh et al., 2019). Similar safety results were reported for other niosome formulations by Alkilani *et al* (Zaid Alkilani et al., 2022), Pourmoghadasiyan *et al* (Pourmoghadasiyan et al., 2022), and Ghafelehbashi *et al (*Ghafelehbashi et al., *2019**)*.

## 4 Conclusions

Microfluidic mixing is an efficient tool that can be used for the preparation of niosomes as a drug delivery system for various molecules such as hydrophilic therapeutics, like atenolol. The type of surfactant and the lipid composition has effects on the physicochemical characteristics. The ability of the niosomes to encapsulate atenolol and to release it was shown to be affected by the niosome composition. Further, the results showed that these atenolol loaded niosomes had a high safety profile on two types of mammalian cells which indicates the ability of using niosomes to control the release of the loaded drug without a significant cytotoxic effect.

## Declaration of Competing Interest

The authors declare that they have no known competing financial interests or personal relationships that could have appeared to influence the work reported in this paper.

## Data Availability

Data will be made available on request.

## References

[bb0005] Abdelkader H., Alani A.W., Alany R.G. (2014). Recent advances in non-ionic surfactant vesicles (niosomes): self-assembly, fabrication, characterization, drug delivery applications and limitations. Drug Deliv..

[bb0010] Aljabali A.A., Obeid M.A. (2020). Inorganic-organic nanomaterials for therapeutics and molecular imaging applications. Nanosci. Nanotechnol. Asia.

[bb0015] Aljabali A.A. (2019). Gold-coated plant virus as computed tomography imaging contrast agent. Beilstein J. Nanotechnol..

[bb0020] Aljabali A.A. (2020). Applications of Nanomaterials in Human Health.

[bb0025] Alyamani H. (2019). Exosomes: fighting cancer with cancer. Ther. Deliv..

[bb0030] Belliveau N.M. (2012). Microfluidic synthesis of highly potent limit-size lipid nanoparticles for in vivo delivery of siRNA. Mol. Ther. Nucl. Acids.

[bb0035] Bhardwaj P. (2020). Niosomes: a review on niosomal research in the last decade. J. Drug Deliv. Sci. Technol..

[bb0040] Carugo D. (2016). Liposome production by microfluidics: potential and limiting factors. Sci. Rep..

[bb0045] Correia M.G.S. (2017). Microfluidic manufacturing of phospholipid nanoparticles: stability, encapsulation efficacy, and drug release. Int. J. Pharmaceut..

[bb0050] El-Assal M. (2017). Proniosomes as nano-carrier for transdermal delivery of atenolol niosomal gel. Int. J. Drug Deliv. Technol..

[bb0055] Gebril A. (2022). Mucosal and systemic immune responses following mucosal immunisation of tetanus toxoid entrapped in lipid nanoparticles prepared by microwave reactor. Eur. J. Pharm. Biopharm..

[bb0060] Ghafelehbashi R. (2019). Preparation, physicochemical properties, in vitro evaluation and release behavior of cephalexin-loaded niosomes. Int. J. Pharmaceut..

[bb0065] Gonzalez Gomez A., Hosseinidoust Z. (2020). Liposomes for antibiotic encapsulation and delivery. ACS Infect. Dis..

[bb0070] Gouda R., Baishya H., Qing Z. (2017). Application of mathematical models in drug release kinetics of carbidopa and levodopa ER tablets. J. Dev. Drugs.

[bb0075] Gregoriadis G. (1973). Drug entrapment in liposomes. FEBS Lett..

[bb0080] Hajizadeh M.R. (2019). In vitro cytotoxicity assay of D-limonene niosomes: an efficient nano-carrier for enhancing solubility of plant-extracted agents. Res. Pharm. Sci..

[bb0085] Jain A., Jain S.K. (2016). In vitro release kinetics model fitting of liposomes: an insight. Chem. Phys. Lipids.

[bb0090] Lo C.T. (2010). Controlled self-assembly of monodisperse niosomes by microfluidic hydrodynamic focusing. Langmuir.

[bb0095] Marianecci C. (2014). Niosomes from 80s to present: the state of the art. Adv. Colloid Interf. Sci..

[bb0100] Maritim S., Boulas P., Lin Y. (2021). Comprehensive analysis of liposome formulation parameters and their influence on encapsulation, stability and drug release in glibenclamide liposomes. Int. J. Pharmaceut..

[bb0105] Meerloo J.V., Kaspers G.J., Cloos J. (2011). Cancer Cell Culture.

[bb0110] Muzzalupo R., Mazzotta E. (2019). Do niosomes have a place in the field of drug delivery?. Expert Opin. Drug Deliv..

[bb0115] Nowroozi F. (2018). Effect of surfactant type, cholesterol content and various downsizing methods on the particle size of niosomes. Iran. J. Pharm. Res. IJPR.

[bb0120] Obeid M.A. (2017). Comparison of the physical characteristics of monodisperse non-ionic surfactant vesicles (NISV) prepared using different manufacturing methods. Int. J. Pharmaceut..

[bb0125] Obeid M.A. (2017). The effects of hydration media on the characteristics of non-ionic surfactant vesicles (NISV) prepared by microfluidics. Int. J. Pharmaceut..

[bb0130] Obeid M.A. (2019). Microfluidic manufacturing of different niosomes nanoparticles for curcumin encapsulation: Physical characteristics, encapsulation efficacy, and drug release. Beilstein J. Nanotechnol..

[bb0135] Obeid M.A. (2020). Examination of the effect of niosome preparation methods in encapsulating model antigens on the vesicle characteristics and their ability to induce immune responses. J. Liposome Res..

[bb0140] Obeid M.A. (2020). Niosome-encapsulated balanocarpol: compound isolation, characterisation, and cytotoxicity evaluation against human breast and ovarian cancer cell lines. Nanotechnology.

[bb0145] Obeid M.A. (2020). Examination of the effect of niosome preparation methods in encapsulating model antigens on the vesicle characteristics and their ability to induce immune responses. J. Liposome Res..

[bb0150] Obeid M.A. (2021). Use of nanoparticles in delivery of nucleic acids for melanoma treatment. Methods Mol. Biol. (Clifton, NJ).

[bb0155] Obeid M.A. (2021). Melanoma.

[bb0160] Pourmoghadasiyan B. (2022). Nanosized paclitaxel-loaded niosomes: formulation, in vitro cytotoxicity, and apoptosis gene expression in breast cancer cell lines. Mol. Biol. Rep..

[bb0165] Sangboonruang S. (2021). Potentiality of melittin-loaded niosomal vesicles against vancomycin-intermediate Staphylococcus aureus and Staphylococcal skin infection. Int. J. Nanomedicine.

[bb0170] Shah H.S. (2020). Preparation and characterization of anticancer niosomal withaferin–a formulation for improved delivery to cancer cells: in vitro, in vivo, and in silico evaluation. J. Drug Deliv. Sci. Technol..

[bb0175] Taymouri S., Varshosaz J. (2016). Effect of different types of surfactants on the physical properties and stability of carvedilol nano-niosomes. Adv. Biomed. Res..

[bb0180] Temprom L. (2022). A novel preparation and characterization of melatonin loaded niosomes based on using a ball milling method. Mater. Today Commun..

[bb0185] Tomeh M.A. (2022). Optimization of large-scale manufacturing of biopolymeric and lipid nanoparticles using microfluidic swirl mixers. Int. J. Pharmaceut..

[bb0190] Whitesides G.M. (2006). The origins and the future of microfluidics. Nature.

[bb0195] Yasamineh S. (2022). A state-of-the-art review on the recent advances of niosomes as a targeted drug delivery system. Int. J. Pharmaceut..

[bb0200] Zaid Alkilani A. (2022). Vitro Studies, Stability Study and Cytotoxicity Study. Nanomaterials.

